# Demographic and radiographic factors for knee symptoms and range of motion in patients with knee osteoarthritis: a cross-sectional study in Beijing, China

**DOI:** 10.1186/s12891-023-06432-8

**Published:** 2023-05-12

**Authors:** Ge Zhou, Minwei Zhao, Xinguang Wang, Xiao Geng, Hua Tian

**Affiliations:** 1 Department of Orthopedics, Peking University Third Hospital, No.49 North Garden Road, Haidian District, Beijing, 100191 China; 2 Engineering Research Center of Bone and Joint Precision Medicine, No.49 North Garden Road, Haidian District, Beijing, 100191 China

**Keywords:** Knee osteoarthritis, Symptoms, Range of motion

## Abstract

**Background:**

Knee osteoarthritis (KOA) causes not only pain, stiffness, and dysfunction of the knee, but also the reduction of the joint range of motion (ROM). This study explored the demographic and radiographic factors for knee symptoms and ROM in patients with symptomatic KOA.

**Methods:**

The demographic variables, Kellgren-Lawrence (KL) grade, and the Western Ontario and McMaster Universities Osteoarthritis Index (WOMAC) of patients with symptomatic KOA recruited in Beijing were collected. The knee ROM of all patients were also measured. We analyzed the influencing factors for WOMAC and ROM using a generalize linear model, respectively.

**Results:**

This study included a total of 2034 patients with symptomatic KOA, including 530 males (26.1%) and 1504 females (73.0%), with a mean age of 59.17 (± 10.22) years. Patients with advanced age, overweight or obesity, a family history of KOA, a moderate-to-heavy manual labor job and use of nonsteroidal anti-inflammatory drugs (NSAIDs) had significantly higher WOMAC and lower ROM (all P < 0.05). The more the comorbidities, the higher the WOMAC (all P < 0.05). Patients with higher education had better ROM than those with only an elementary education(β = 4.905, P < 0.05). Compared with those KL = 0/1, the WOMAC of patients whose KL = 4 were higher (β = 0.069, P < 0.05), but the WOMAC of those KL = 2 were lower (β = -0.068, P < 0.05). ROM decreased with the increase of KL grade (all P < 0.05).

**Conclusions:**

KOA patients with advanced age, overweight or obesity, a family history of KOA in first-degree relatives, a moderate-to-heavy manual labor job tended to have more severe clinical symptoms and worse ROM. Patients with more severe imaging lesions tend to have poorer ROM. Symptom management measures and regular ROM screening should be taken early to these people.

## Background

Osteoarthritis (OA) is a joint degenerative disease with joint pain and restricted mobility as the main manifestations, which causes a heavy social and financial burden. In 2020, KOA was estimated to affect approximately 650 million people worldwide [1]. It is widely accepted that the burden of KOA will continue to increase with population ageing across the world [2].

Pain, stiffness, and dysfunction of the knee caused by symptomatic KOA seriously affect the quality of life of patients. The term ‘symptomatic’ signifies the group of individuals seeking health care for their symptoms and who thereby differ from persons with risk factors for OA but without symptoms. Risk factors for KOA have been widely studied, including female sex, increasing age [3], obesity [4], genetic factors [5], repetitive joint use through occupation [6], etc. However, the rarely reported influencing factors for KOA symptoms may be different from the risk factors for KOA. Therefore, it is important to clarify the demographic and radiographic factors for KOA symptoms and formulate population-specific treatment strategies to reduce the disease burden.

Reduced knee ROM is an important clinical manifestation of KOA, which restricts mobility and interfere with basic activities of daily life such as walking and standing [7]. Lost knee extension is a risk factor for total knee arthroplasty (TKA) [8, 9]. However, accurately measuring ROM can be time consuming, and frequently-used clinical OA scales often do not include measuring joint ROM [10]. As a result, the detection, monitoring, and therapeutic measures needed to optimize the care of preventing ROM from decreasing may be ignored. Once the knee ROM has reduced, it will be difficult to reverse with treatment. To our knowledge, only a handful of studies reported that demographic, articular, and clinical factors seem to have influence on reduced ROM, such as age [11], body mass index (BMI) [12, 13], radiographic joint space narrowing (JSN) and osteophytes [13, 14].

This study analyzed the demographic and radiographic factors for knee symptoms and ROM in patients with symptomatic KOA, to identify the targeted population for disease intervention and provide reference for formulating population-specific prevention and treatment strategies for KOA.

## Methods

### Study design

This multicenter cross-sectional study was carried out from December 2017 to November 2018 and involved five clinical centers: Peking University Third Hospital, Peking University First Hospital, Peking University People’s Hospital, Beijing Friendship Hospital, and China-Japan Friendship Hospital. All of the five centers are large tertiary general hospitals, serving patients at all stages of KOA disease.

### Participants

Patients with KOA in the outpatient clinic of the aforementioned five centers were recruited. The inclusion criteria were (1) patients with primary KOA diagnosed according to the diagnostic criteria revised by the Chinese Orthopaedic Association in 2007 (Table 1); and (2) patients with a household registration or permanent residence in Beijing. The exclusion criteria were (1) knee joint pain caused by diseases other than KOA (e.g., infection, hip joint disease, and lumbar disk disease); and (2) secondary KOA (e.g., traumatic OA, rheumatoid arthritis, and ankylosing spondylitis).

**Table 1:** Diagnostic criteria for KOA according to the Chinese Medical Association Orthopaedic Branch (2007 revision)

1. Repeated knee pain in the last month
2. X-ray film (standing or weight-bearing position) shows narrowing of joint space, subchondral bone sclerosis and/or cystic changes, joint edge formation
3. Joint fluid (at least two times) clear, viscous, WCC < 0.002 × 10^9^/L
4. Middle-aged or elderly patients (≥ 40 years old)
5. Morning stiffness ≤ 3 min
6. Bone friction sound (feeling) during activity

Patients with 1 + 2 or 1 + 3 + 5 + 6 or 1 + 4 + 5 + 6 can be diagnosed with KOA

KOA: knee osteoarthritis; WCC: white cell count

### Demographic and radiographic variables

Demographic variables of the patients including sex, age, body mass index (BMI), physical labor intensity of occupation, education level, KOA history in first-degree relatives (parents and siblings), and comorbidities were collected in the form of a questionnaire. Among those variables, the recommended cutoff values of BMI < 24 kg/m^2^, 24.0–27.9 kg/m^2^, and ≥ 28.0 kg/m^2^ for Chinese adults [15] were used to classify patients as underweight or normal weight, overweight, and obese, respectively. An educational level of ≤ 9 years, 10–12 years, and > 12 years were considered elementary education, secondary education, and higher education, respectively. The Charlson comorbidity index (CCI) was used to quantitively evaluate the number and severity of comorbidities about the patients [16]. The use of NSAIDS in the last one month was also recorded. Each patient was subjected to X-ray examination of the knee joint. We used the KL classification proposed by Kellgren and Lawrence [17] to define the severity of imaging lesions.

### Outcome measurement

The Western Ontario and McMaster Universities Osteoarthritis Index (WOMAC) of each patient was collected in the form of a questionnaire [18]. The WOMAC has been extensively used to measure pain, stiffness and physical disability of knee joints in patients with KOA. The higher the WOMAC score, the worse the health status of the patient. This scoring system has been found to have good reliability [19].

Each patient was asked to maximally extend the knee and then maximally flex the knee for the ROM evaluation. The goniometer was positioned with its center fulcrum over the lateral epicondyle of the femur. The proximal arm was aligned with the lateral midline of the femur, using the greater trochanter for reference, and the distal arm was aligned with the lateral midline of the fibula, using the lateral malleolus and fibular head for reference. Measurements were made three times, taking the average value to analyze data. ROM was recorded as the arc of active motion from extension to flexion.

### Statistical analyses

SPSS 24.0 software package (IBM, Armonk, NY, USA) was used for the statistical analyses. All indicators were tested for normality and analysis of homogeneity of variance. The non-normally distributed data (WOMAC) were presented by the median (interquartile range (p_25_, p_75_)), while the normally distributed data (ROM) were denoted by $\stackrel{-}{x}$± s. Intergroup comparisons were performed using the Mann-Whitney U test or the Kruskal-Wallis H test for the WOMAC, and t test or univariate analysis of variance for the ROM. All demographic and radiographic variables were included in the generalized linear model (GLM) to evaluate the risk factors for WOMAC and ROM. We used gamma distribution for the WOMAC in the GLM. For the normally distributed ROM, we used linear regression. The results were presented as regression coefficient β and 95% confidence interval (CI). *P* < 0.05 considered statistically significant.

## Results

### Participant characteristics

This study initially enrolled 2066 patients with symptomatic KOA in Beijing, of whom 32 had incomplete data and were excluded, resulting in 2034 patients enrolled in this study. Of all included patients, there were 530 males (26.1%) and 1504 females (73.9%), with a mean age of 59.17 (± 10.22) years. There were 941 overweight patients (46.3%) and 335 obese patients (16.5%); The first-degree relatives of 808 patients (39.7%) had a history of KOA; 876 patients had higher-education qualifications (43.1%). There were 126 patients (6.2%) engaged in moderate-to-heavy manual labor jobs. A total of 360 patients (17.7%) had a CCI of 1 and 232 patients (11.4%) had a CCI ≥ 2. In the last one month, 649 patients (31.9%) had used NSAIDS because of knee pain. The number of patients with a KL = 0, 1, 2, 3, 4 was 182 (8.9%), 363 (17.8%), 748 (36.8%), 595 (29.3%), 146 (7.2%), respectively. Table 2 shows the demographic and radiographic data of the patients.

### Analysis of influencing factors for symptoms and ROM in patients with KOA

Univariate analysis of the data in Table 2 showed that the WOMAC and ROM were significantly different among the patients in different age groups, with different sex, BMI, physical labor intensity of occupation, education level, history of KOA in first-degree relatives, CCI, usage of NSAIDS and KL grades (all P < 0.05).

**Table 2:** Intergroup comparisons of WOMAC and ROM in patients with KOA (n = 2034)

	n (%)	WOMAC		ROM	
		median (p_25_, p_75_)	P	$\stackrel{-}{x}$±*s*	P
Sex			< 0.001		< 0.001
Male	530 (26.1)	37.0 (30.0, 45.0)		125.2 ± 11.4	
Female	1504 (73.9)	41.0 (32.0, 48.0)		122.8 ± 13.3	
Age (yeas)			< 0.001		< 0.001
< 55	607 (29.8)	35.0 (30.0, 42.4)		127.4 ± 11.7	
55–64	696 (34.2)	40.0 (32.0, 49.0)		123.4 ± 11.9	
≥ 64	731 (36.0)	42.2 (34.0, 51.0)		120.1 ± 13.8	
BMI (kg/m2)			< 0.001		< 0.001
< 24	758 (37.2)	36.5 (30.0, 45.0)		126.0 ± 12.1	
24–27.9	941 (46.3)	40.0 (32.0, 48.0)		122.6 ± 12.5	
≥ 28	335 (16.5)	42.4 (36.0, 52.0)		119.9 ± 14.3	
History of KOA in first-degree relatives			< 0.001		< 0.001
No	1226 (60.3)	38.0 (31.0, 46.0)		124.8 ± 13.6	
Yes	808 (39.7)	42.0 (33.0, 49.0)		122.5 ± 12.3	
Years of education (years)			0.046		< 0.001
≤ 9	611 (30.0)	40.0 (32.0, 50.0)		120.5 ± 12.4	
10–12	547 (26.9)	41.0 (32.0, 49.0)		121.9 ± 13.7	
≥ 13	876 (43.1)	39.0 (31.0, 42.0)		126.4 ± 12.0	
Physical labor intensity of occupation			< 0.001		0.001
Sedentary	936 (46.0)	40.0 (32.0, 46.0)		124.4 ± 12.8 122.3 ± 13.0	
Mild manual labor	972 (47.8)	39.0 (31.0, 48.0)			
Moderate-to-heavy manual labor	126 (6.2)	42.4 (37.0, 53.0)		125.2 ± 12.3	
CCI			< 0.001		< 0.001
0	1442 (70.9)	38.0 (31.0, 46.0)		124.2 ± 12.4 121.9 ± 14.0	
1	360 (17.7)	42.4 (33.0, 50.0)			
≥ 2	232 (11.4)	42.4 (34.0, 56.0)		120.9 ± 13.7	
Use of NSAIDS			< 0.001		< 0.001
0	1385 (68.1)	37.0 (30.0, 44.0)		124.9 ± 12.4	
1	649 (31.9)	42.4 (36.0, 54.0)		120.4 ± 12.3	
KL grade			< 0.001		< 0.001
0/1	545 (26.7)	39.5 (31.0, 45.0)		126.7 ± 11.5	
2	748 (36.8)	37.0 (31.0, 45.0)		121.9 ± 11.3	
3	595 (29.3)	40.0 (32.0, 49.0)		119.3 ± 12.7	
4	146 (7.2)	42.7 (40.0, 60.0)		118.7 ± 16.0	

BMI: Body mass index; CCI: Charlson comorbidity index; KL: Kellgren - Lawrence; WOMAC: The Western Ontario and McMaster Universities Osteoarthritis Index; ROM: range of motion

Incorporation of all of the above variables into a model of WOMAC and ROM (Table 3) showed patients with age ≥ 64, overweight or obesity, history of knee OA in first-degree relatives, moderate-to-heavy manual labor job and use of NSAIDS had more severe symptoms and worse ROM (all P < 0.05). Compared to patients aged < 55, those aged 55–64 had higher WOMAC (β = 0.078, P < 0.05), while the ROM between the two groups had no statistical difference (β = -0.706, P > 0.05). ROM of patients with higher education was significantly higher than those with elementary education (β = 4.905, P < 0.05). WOMAC increased with CCI (all β > 0, all P < 0), but groups of CCI ≥ 2 did not show worse ROM compared to group of CCI = 1 (all P > 0.05). ROM was negatively correlated with KL (all β < 0, all P < 0.05), while WOMAC was not positively correlated with KL. Compared with individuals whose KL = 0 or 1, those KL = 2 had lower WOMAC (β = -0.068, P < 0.05), but those KL = 4 had higher WOMAC (β = 0.069, P < 0.05).

**Table 3:** Analysis of influencing factors for WOMAC and ROM in patients with KOA (n = 2034)

	WOMAC		ROM	
	β (95%CI)	P	β (95%CI)	P
Sex				
Male	Ref		Ref	
Female	0.026 (-0.010, 0.061)	0.162	-1.248 (-2.834, 0.338)	0.123
Age (yeas)				
< 55	Ref		Ref	
55–64	0.078 (0.037, 0.118)	< 0.001	-0.706 (-2.507, 1.095)	0.442
≥ 64	0.093 (0.052, 0.134)	< 0.001	-2.370 (-4.213, -0.526)	0.012
BMI (kg/m2)				
< 24	Ref		Ref	
24–27.9	0.051 (0.018, 0.083)	< 0.001	-1.839 (-3.276, -0.402)	0.012
≥ 28	0.105 (0.059, 0.151)	0.002	-4.977 (-7.018, -2.936)	< 0.001
History of KOA in first-degree relatives				
No	Ref		Ref	
Yes	0.059 (0.028, 0.090)	< 0.001	-1.721 (-3.101, -0.340)	0.015
Years of education (years)				
≤ 9	Ref		Ref	
10–12	-0.023(-0.062, 0.016)	0.248	1.443 (-0.280, 3.165)	0.101
≥ 13	0.010 (-0.029, 0.050)	0.603	4.905 (3.182, 6.628)	< 0.001
Physical labor intensity of occupation				
Sedentary	Ref		Ref	
Mild manual labor	0.018(-0.014, 0.051)	0.268	-1.199 (-2.608, 0.211)	0.096
Moderate-to-heavy manual labor	0.108 (0.039, 0.177)	0.002	-4.381 (-7.433, -1.328)	0.005
CCI				
0	Ref		Ref	
1	0.049 (0.011, 0.088)	0.013	-1.628 (-3.331, 0.074)	0.061
≥ 2	0.099 (0.050, 0.147)	< 0.001	-0.604 (-2.746, 1.538)	0.580
Use of NSAIDS				
0	Ref		Ref	
1	0.111(0.079, 0.143)	< 0.001	-1.586 (-3.010, -0.163)	0.029
KL grade				
0/1	Ref		Ref	
2	-0.068 (-0.106, -0.029)	0.001	-2.934 (-4.650, -1.218)	0.001
3	-0.032(-0.074, 0.010)	0.135	-4.938 (-6.801, -3.075)	< 0.001
4	0.069(0.003, 0.134)	0.039	-5.058 (-7.921, -2.195)	0.001

BMI: Body mass index; CI: Confidence interval; CCI: Charlson comorbidity index; KL: Kellgren – Lawrence; WOMAC: The Western Ontario and McMaster Universities Osteoarthritis Index; ROM: range of motion

## Discussion

To our knowledge, this is the first study to analyze the demographic and radiographic factors for symptoms and ROM in patients with KOA in China. Some factors are nearly the same for both of knee symptoms and ROM: advanced age, overweight or obesity, a family history of KOA in first-degree relatives, a moderate-to-heavy manual labor job, which are also the risk factors for KOA. Other factors for symptoms and ROM seem to be different, such as education level, comorbidities and KL grades. Use of NSAIDs is significantly associated with WOMAC and ROM.

Age and BMI are two of the most significant risk factors for KOA. From our results, we noticed that compared with individuals aged < 55, those aged 55–64 manifested more severe symptoms but no worse ROM. This may be interpreted as a later onset of ROM decline than clinical symptoms for KOA. Flexion contracture usually appears in the late stages of OA. Therefore, Early intervention is important for those who have symptoms but have not yet experienced a decline in ROM. As for BMI, the increase of mechanical load caused by overweight or obesity and the inflammatory load caused by cytokines produced by adipose tissue both lead to the aggravation of knee pain [20]. Besides, the thick, redundant fatty deposits covered around the knee will prevent the joint from achieving full flexion [12]. On the other hand, the restriction of movement caused by KOA may lead to weight gain, thereby forming a vicious cycle. Thus, weight loss is strongly recommended for overweight or obese KOA patients [21].

KOA is not only affected by genetic factors, but also by acquired factors such as occupation and education. A prospective cohort study [22] showed that offspring with a family history of KOA have an increased risk of worsening knee pain, which is independent of structural factors, suggesting that genetic factors may be involved in the pathogenesis of knee pain. Many occupational factors, such as kneeling, squatting, carrying heavy objects, and climbing stairs are significantly associated with KOA [23]. In this study, we found that individuals with higher education had better knee ROM, which may be attributed to their better awareness of OA management. For those with genetic predisposition and occupational risk factors, medical and health institutions should actively carry out health education and emphasize the importance of changing life and working style.

Comorbidity is common among people with OA. Previous studies have reported that 31% of OA patients have multiple chronic diseases [24], the most common of which is metabolic syndrome [25]. KOA is associated with a significantly increased risk of cardiovascular events, likely due to the limited activity and lack of exercise in patients that aggravate the decline of cardiopulmonary function [26]. Peptic ulcer disease and renal disease are also related to OA [27]. We found that comorbidities seem to have a greater effect on symptoms than ROM. Comorbidity has been shown to be closely associated with increased pain among OA patients [28]. One possible explanation is that impairment due to one disease may exacerbate that due to another: for example, the pain associated with OA may be exacerbated by diabetic neuropathy. Additional factors such as psychological impairment caused by comorbidities may also play a role in the burden on OA symptoms. However, ROM is rarely affected by these factors, it is more closely related to the degree of knee joint lesions.

In this research, there is no significant negative correlation between WOMAC and KL grade. Studies have shown that radiographic evidence of knee damage predisposes to knee pain, but the severity of the radiographic findings is rather weakly associated with the severity of the symptoms [29, 30]. At the early symptomatic stage of the disease, there are often no or only limited radiographically detectable structural changes (KL grade 0–1) [31]. Ersoz et al. [32] found significant negative correlations between ROM and KL radiographic scores of knee joint. We came to a similar conclusion. ROM decline and radiographic changes may be a reciprocal causation. Decreased knee ROM limits the stress area and increases local hydrostatic pressures, leading to chondrocyte apoptosis and further cartilage degeneration [33]. Conversely, structural changes associated with KOA, including osteophytes and JSN, may contribute to loss of ROM by mechanically blocking knee extension or flexion [13, 14]. Population with risk factors for developing KOA should be subjected to regular ROM screening.

There are some limitations to our study. First, no causal conclusions can be inferred because the study was cross-sectional in design. Second, the location of osteophytes and JSN on radiographs was not documented in detail, which may affect flexion ROM and extension ROM by different mechanisms. Third, comorbidities of patients and KOA in first-degree relatives were based on self-report, the diagnosis of which may not be accurate.

## Conclusions

There are both similarities and differences in the factors affecting knee symptoms and ROM. People with advanced age, overweight or obesity, a family history of KOA in first-degree relatives, a moderate-to-heavy manual labor job are the targeted population of symptom prevention and should be subjected to early ROM screening. ROM decline are more closely related to imaging changes than symptom severity. We hope this study will provide a reference basis for subsequent formulation of prevention and therapeutic strategies for KOA.

## Data Availability

The datasets analyzed during the current study are not publicly available because the follow-up work of Chinese primary knee osteoarthritis progression cohort (CPKOPC) has not been completed yet, but are available from the corresponding author on reasonable request.

## Abbreviations

KOA: Knee osteoarthritis
OA: Osteoarthritis
ROM: Range of motion
KL: Kellgren-Lawrence
WOMAC: The Western Ontario and McMaster Universities Osteoarthritis Index
TKA: Total knee arthroplasty
BMI: Body mass index
JSN: Joint space narrowing
WCC: White cell count
CCI: Charlson comorbidity index
CI: Confidence interval
